# Dramatic clearance of extensive psoriasis in a pediatric patient treated with upadacitinib: A case report

**DOI:** 10.1177/2050313X251387391

**Published:** 2025-12-11

**Authors:** Jenna Mistry, Michal Moshkovich, Maxwell Sauder, Cathryn Sibbald

**Affiliations:** 1 Temerty Faculty of Medicine, University of Toronto, ON, Canada; 2 Princess Margaret Hospital Cancer Centre, Toronto, ON, Canada; 3 Division of Dermatology, Department of Medicine, University of Toronto, ON, Canada; 4 Division of Dermatology, Department of Paediatrics, The Hospital for Sick Children, Toronto, ON, Canada

**Keywords:** atopic dermatitis, JAK inhibitors, pediatric, psoriasis, upadacitinib

## Abstract

We present a unique case of an 8-year-old boy with severe, treatment-refractory palmoplantar psoriasis and genital involvement, unresponsive to nine systemic agents, including multiple biologics and TYK2 inhibition with deucravacitinib. Following years of debilitating symptoms and functional impairment, he achieved near-complete resolution within 2 weeks of initiating upadacitinib monotherapy. This dramatic and sustained response highlights the potential role of JAK1 inhibition in modulating complex inflammatory pathways in pediatric psoriasis, particularly in challenging anatomic sites and refractory cases.

## Introduction

Psoriasis is a chronic inflammatory skin condition that commonly presents on the knees, elbows, trunk, and scalp but can involve the palmoplantar surfaces. FDA-approved treatments for pediatric psoriasis are limited, often requiring off-label use of systemic agents. Upadacitinib, an oral selective and reversible Janus kinase (JAK) inhibitor approved for atopic dermatitis (AD) in patients older than 12, has shown efficacy in psoriatic arthritis and plaque psoriasis in adults.^1,2^ Upadacitinib inhibits JAK phosphorylation and displays more selectivity toward JAK1 to reduce inflammation. We report a case of severe palmoplantar psoriasis in an 8-year-old child that achieved near-complete resolution on upadacitinib.

## Case report

**Figure 1.**
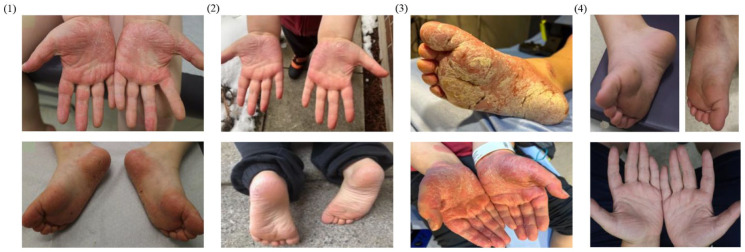
Chronologic improvement of palmoplantar psoriasis with systemic therapies. (1) Baseline palmoplantar surfaces before initiation of systemic treatment. (2) Palmoplantar surfaces after 3 months of ustekinumab usage. (3) Acute flare requiring hospitalization with widespread plaques. (4) Completely resolved flares and plaques after use of upadacitinib for 3 weeks.

**Figure 2.**
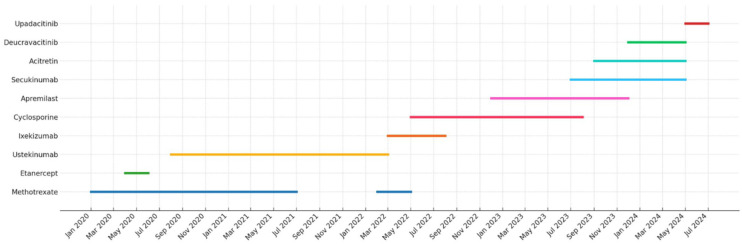
Individualized treatment course for chronic palmoplantar psoriasis.

In 2020, an 8-year-old boy presented with severe chronic palmoplantar psoriasis with genital involvement since age 3, worsening over the previous year. A biopsy was completed by his community dermatologist that was consistent with psoriasis. Figures 1 and 2 demonstrate his clinical presentation and treatment history. On initial presentation, plaques involved his axillae, scrotum, and buttocks, along with deep, painful fissures on his palms and soles, limiting mobility. His nails had deep pitting with classic subungual hyperkeratosis and onycholysis. Treatment with topical clobetasol and combination betamethasone diproprionate/calcipotriol led to only partial improvement on his trunk. Methotrexate (MTX; 10 mg) with folic acid (1 mg) supplementation and topical crisaborole were initiated (Table 1). Topical crisaborole was chosen based on a study demonstrating benefit in inverse psoriasis.^3^

**Table 1.** Longitudinal palmoplantar psoriasis medication management timeline.

Medication	Start date	Dose and duration	Comments
Methotrexate	January 2020	Patient was 40 kg. Weekly dose was weight-based (0.5 mg/kg/week) 20 mg orally weekly (0.5 mg/kg) × 5 months Then 15 mg orally weekly × 7 months (with etanercept and ustekinumab) Then 10 mg orally weekly × 5 months Then 15 mg orally weekly × 3 months	Elevated liver enzymes, low density lipoprotein and triglycerides at higher dose Liver enzymes stabilized on lower dose
Etanercept	April 2020	0.8 mg/kg/week SC (32 mg) × 10 weeks	Incontinence and neurologic symptoms (saddle anesthesia), not confirmed to be directly related
Ustekinumab	August 2020	45 mg SQ q12 weeks × 14 months Then 45 mg q8 weeks × 6 months	Initial response then flared despite decrease in dose interval
Ixekizumab	March 2022	80 mg q4weeks × 6 months	Painful injections (despite Ativan + non-pharmacologic measures)
Cyclosporine	May 2022	5 mg/kg/day orally divided twice daily × 16 months	Increased low density lipoprotein and triglycerides
Apremilast	December 2022	15 mg orally twice daily × 1 month Then 15 mg daily × 12 months	Nausea and vomiting with twice daily dosing, subsided with once daily dosing
Secukinumab	July 2023	150 mg SC q2 weeks × 8 Then q4 weeks × 10 months	Tolerated well but no benefit
Acitretin	September 2023	10 mg orally daily × 3 months Then 25 mg daily × 6 months	Increased low density lipoprotein and triglycerides
Deucravacitinib	December 2023	6 mg orally daily × 6 months	Tolerated well but no benefit
Upadacitinib	May 2024	15 mg orally daily × 3 months	Complete resolution of all psoriasis plaques and fissures

After 4 months, there was limited improvement, and etanercept was started at 0.8 mg/kg weekly with an increase of MTX to 15 mg weekly. He continued to have significant palmoplantar pain. In July 2020, he developed urinary incontinence and was hospitalized for saddle anesthesia and severe constipation, attributed to his avoidance of walking to the bathroom due to pain. Saddle anesthesia was documented by emergency department staff clinically and with normal imaging.

Etanercept was stopped, and ustekinumab was added to MTX and topical mometasone. This resulted in moderate improvement, with a reduction in pain and pruritus. MTX was decreased, and his BSA improved to 3%, with nearly clear hands and feet, and only small plaques on his knees.

In July 2021, MTX was discontinued, in part due to increased liver enzymes, and he remained on ustekinumab and topical steroids. Unfortunately, his psoriasis worsened with new hyperkeratotic plaques with fissuring of the palms and soles. Ustekinumab dosing was increased to every 8 weeks, and topical halobetasol/tazarotene was prescribed.

Despite these changes, his psoriasis progressed to his back, chest, and inner thighs. Liquor carbonis detergens 10% in halobetasol 0.05% ointment was started, then MTX (15 mg) with folic acid was restarted given liver enzymes stabilization. Patient stopped attending school due to pain and was crawling at home. He had plaques with thickened scale and dried hemorrhagic crust within demarcated fissures.

At this point, cyclosporine (110 mg twice daily) was started, and ustekinumab stopped, with subsequent initiation of Ixekizumab (80 mg every 4 weeks). While pain and pruritus in his palms and soles persisted, he started to bear weight. Apremilast (15 mg twice daily) was added but quickly dose reduced to once daily due to gastrointestinal side effects. With this, most of his psoriasis cleared, leaving only a small area on his palms.

An attempt at tapering cyclosporine resulted in a palmoplantar flare, and the patient refused ixekizumab injection after 6 months of use due to significant ongoing pain despite interventions (anxiolytics, topical anesthesia). Secukinumab was added to his regimen 2 months later, and cyclosporine was discontinued. Acitretin (10 mg/day), halobetasol/tazarotene lotion, and Roflumilast cream were added. Psoriasiform plaques persisted and worsened on his wrists, shins, palms, and soles, with fissuring on the soles impairing mobility.

In November 2023, after failing several systemic agents, the patient was hospitalized for pain control and refractory disease. His disease was widespread and severe, and pain was managed with a multi-drug regimen including gabapentin. Deucravacitinib (6 mg) was introduced on a compassionate basis. While fissuring on the soles improved, extensive plaques on the body persisted.

At a clinic re-assessment in April 2024, all current treatments (secukinumab, deucravacitinib, and acitretin) were stopped due to inefficacy and a concern that some could be inducing paradoxical worsening. A trial of upadacitinib (15 mg) monotherapy was offered. Within 2 weeks, the patient achieved near-complete clearance of palmoplantar psoriasis and nail pitting. Pain and fissures resolved, allowing the patient to return to normal activities and stop the gabapentin. At 2 and 6-month follow-up visits, his skin remained clear, and he reported improved quality of life.

## Discussion

FDA-approved systemic treatments for pediatric psoriasis include biologics targeting inflammatory pathways but are fewer in number than adult options.^4^ Other systemic drugs (acitretin, methotrexate, cyclosporine) and phototherapy are used off-label for treatment.^5^

We report an impressive case of complete clearance of severe inverse psoriasis in an adolescent with upadacitinib. The patient did not show any features of AD and initially cleared with etanercept, which would not be expected with AD. Additionally, an autoinflammatory genetic panel revealed no variants, ruling out an underlying genetically mediated syndrome. Several case reports report positive outcomes for patients with refractory psoriatic arthritis and psoriasis treated with upadacitinib (Table 2). One study suggests that upadacitinib may be more effective for psoriasis when compared to alternative JAK inhibitors due to its selectivity for JAK1.^6^ Coexisting alopecia areata and AD may also improve with upadacitinib.^6–9^ Most patients reported significant response after a month of use with complete or near complete resolution in 3–5 months.^6–14^ Patients were able to continue treatment with no recurrence or adverse events, however, follow-up in most case reports stopped at 32 weeks.

**Table 2.** Characteristics of patients with psoriasis treated with upadacitinib.

Study	Age	Sex	Diagnosis	Previously tried therapies	Daily dose (mg)	Duration (months)	Efficacy	Side effects
Perricone et al.^6^	36	F	Psoriatic arthritis, alopecia universalis	Methotrexate, leflunomide, sulfasalazine, abatacept, golimumab, baricitinib, infliximab, methylprednisolone, hydroxychloroquine	15	3	Partial resolution, no recurrence	None
Tsunoda et al.^7^	25	M	Psoriatic arthritis	Dupilumab, cyclosporine, ixekizumab, prednisolone, baricitinib	30	4	Partial resolution, no recurrence	None
Gargiulo et al.^8^	12	M	Psoriasis, atopic dermatitis	Ustekinumab, dupilumab	15	13	Complete resolution	None
	39	M	Psoriasis, atopic dermatitis	Cyclosporine, brodalumab	15	9	Complete resolution	None
	50	F	Psoriasis, psoriatic arthritis, atopic dermatitis	Salazopyrin, methotrexate, ustekinumab, secukinumab, apremilast	15	8	Complete resolution	None
	42	F	Psoriasis, atopic dermatitis	Acitretin, methotrexate, adalimumab, risankizumab, dupilumab	30	15	Complete resolution	None
Ch’en et al.^9^	50	M	Psoriasis, atopic dermatitis	Topical steroids, secukinumab, tildrakizumab, ixekizumab, methotrexate, intramuscular triamcinolone	15	1 week	Complete resolution	None
	45	M	Psoriasis	Methotrexate, certolizumab, dupilumab	15	2	Complete resolution	None
Graceffa et al.^11^	55 (*n* = 14)	5F, 9M	Psoriatic arthritis	Not reported	15	3	Partial resolution	None
Su et al.^12^	66	M	Psoriasis vulgaris, bullous pemphigoid	Methylprednisolone sodium succinate, prednisone, dupilumab	15	3	Complete resolution, no recurrence	None
Wang et al.^13^	13	F	Psoriasis	Not reported	15	5	Complete resolution	None
Wang et al.^13^	21	M	Pustular psoriasis	Acitretin, methotrexate	15	3	Complete resolution	None

AD is a very common condition causing dry, pruritic, and inflamed skin. Upadacitinib can manage these symptoms, with previous case reports demonstrating effectiveness in patients with concurrent skin conditions.^15–27^ In a 52-week phase III randomized controlled trial, adults and adolescents with moderate to severe AD saw significant improvement in symptoms when treated with upadacitinib 15 or 30 mg.^28^ By week 16, upadacitinib patients experienced greater improvements than those receiving placebo. Early improvements in itch, pain, sleep, quality of life, and mental health were maintained until week 52. Concurrent diseases in patients with AD, such as vitiligo, psoriasis, and alopecia areata showed improvement with upadacitinib.^6,7,18,20,23,24^ Paradoxical psoriasis has also been reported after secukinumab therapy, and two case reports demonstrated successful treatment with upadacitinib.^29,30^

Our patient responded exceptionally well to treatment with upadacitinib. We believe this supports its use for select patients with refractory disease and with palmoplantar involvement. It may also have specific benefits in those with possible paradoxical psoriasis secondary to biologics.
